# Increased diagnostic yield in a cohort of hearing loss families using a comprehensive stepwise strategy of molecular testing

**DOI:** 10.3389/fgene.2022.1057293

**Published:** 2022-12-07

**Authors:** Beiping Zeng, Hongen Xu, Yanan Yu, Siqi Li, Yongan Tian, Tiandong Li, Zengguang Yang, Haili Wang, Guangke Wang, Mingxiu Chang, Wenxue Tang

**Affiliations:** ^1^ BGI College and Henan Institute of Medical and Pharmaceutical Sciences, Zhengzhou University, Zhengzhou, China; ^2^ National Health Commission Key Laboratory of Birth Defects Prevention, Henan Key Laboratory of Population Defects Prevention, Henan Institute of Reproduction Health Science and Technology, Zhengzhou, China; ^3^ Precision Medicine Center, Academy of Medical Science, Zhengzhou University, Zhengzhou, China; ^4^ The Research and Application Center of Precision Medicine, The Second Affiliated Hospital of Zhengzhou University, Zhengzhou, China; ^5^ Department of Physiology and Neurobiology, Academy of Medical Science, Zhengzhou University, Zhengzhou, China; ^6^ College of Public Health, Zhengzhou University, Zhengzhou, China; ^7^ Department of Otorhinolaryngology Head and Neck Surgery, Henan Provincial People’s Hospital, Zhengzhou, China

**Keywords:** hearing loss, deafness, molecular diagnosis, stepwise strategy, GJB2 exon 1, STRC gene

## Abstract

Hearing loss is one of the most common sensory disorders in humans. This study proposes a stepwise strategy of deafness gene detection using multiplex PCR combined with high-throughput sequencing, Sanger sequencing, multiplex ligation-dependent probe amplification (MLPA), and whole-exome sequencing (WES) to explore its application in molecular diagnosis of hearing loss families. A total of 152 families with hearing loss were included in this study, the highest overall diagnosis rate was 73% (111/152). The diagnosis rate of multiplex PCR combined with high-throughput sequencing was 52.6% (80/152). One families was diagnosed by Sanger sequencing of *GJB2* exon 1. Two families were diagnosed by MLPA analysis of the *STRC* gene. The diagnosis rate with additional contribution from WES was 18.4% (28/152). We identified 21 novel variants from 15 deafness genes by WES. Combining WES and deep clinical phenotyping, we diagnosed 11 patients with syndromic hearing loss (SHL). This study demonstrated improved diagnostic yield in a cohort of hearing loss families and confirmed the advantages of a stepwise strategy in the molecular diagnosis of hearing loss.

## Introduction

Hearing loss is the most common sensory disorder in humans, with an estimated prevalence of 1–3 in every 1,000 newborns worldwide (Morton and Nance, 2006). According to the World Report on Hearing by the World Health Organization in 2021, more than 5% of the world’s population (about 466 million people) suffer from hearing loss (https://www.who.int/news-room/fact-sheets/detail/deafness-and-hearing-loss). In China, around 30, 000 babies with congenital hearing impairment are born every year (Dai et al., 2006). It is estimated that genetic factors account for more than 60% of cases of hearing loss (Marazita et al., 1993; Morton and Nance, 2006). The inheritance patterns of deafness include autosomal recessive (80%), autosomal dominant (15%–20%), sex chromosome-linked (1%), and mitochondrial inheritance (1%) (Dai and Yuan, 2017). Hereditary hearing loss is a highly genetically heterogeneous disorder (Brownstein et al., 2012) that can be divided into syndromic hearing loss and non-syndromic hearing loss (NSHL), among which NSHL is the predominant type accounting for ∼70% of cases (Morton and Nance, 2006). To date, more than 400 syndromes related to hearing loss have been found (Alford et al., 2014), and over 110 NSHL genes have been identified (https://hereditaryhearingloss.org/).

Molecular epidemiological studies in China have identified several common deafness genes, such as *GJB2*, *SLC26A4*, and *MT-RNR1*, which account for 30%–50% of congenital hearing loss cases (Yuan et al., 2009; Du et al., 2014). In China, the ratio of patients carrying monoallelic variants in the coding exons of *GJB2* is 6.1% (Dai et al., 2009). However, few studies have examined the non-coding exon 1 of *GJB2* in Chinese patients with hearing loss. In 2010, Yuan et al. (2010) showed that testing for the NM_004004.6 (*GJB2*): c.-23 + 1G>A variant explained deafness in 1.89% (4/212) of Chinese patients with *GJB2* monoallelic variants. In 2020, Yu et al. (2020) screened 1, 852 Chinese patients with deafness by Sanger sequencing in the *GJB2* coding exon and flanking regions as well as the non-coding exon 1 and its flanking splice sites. The results showed that 475 patients had biallelic variants, one of whom carried a c.-23 + 1G>A (1/475, 0.2%) variant. Therefore, Sanger sequencing of *GJB2* exon 1 should be included in routine testing of patients with *GJB2* monoallelic pathogenic variants.

Recently, the *STRC* gene has been suggested to be an important molecular cause of mild-to-moderate deafness (Shearer et al., 2014; Plevova et al., 2017; Yokota et al., 2019). In 2019, Yokota et al. (2019) investigated the frequency of *STRC* deletions in the Japanese population. This study showed that the prevalence of *STRC* homozygous deletions was 1.7% (17/1,025) in the overall population with hearing loss, and 4.3% (17/398) among patients with mild-to-moderate hearing loss. In 2020, Kim et al. (2020) showed that approximately two-thirds (52/83, 62.7%) of mild-to-moderate sensorineural hearing loss (SNHL) have a clear Mendelian genetic etiology, with *STRC*-related deafness (29/83, 34.9%) being the most prevalent. These studies highlight the *STRC* gene as a major cause of mild-to-moderate hearing loss.

Due to the high genetic heterogeneity of hearing loss, it is necessary to develop a comprehensive and appropriate diagnostic strategy for patients. Whole-exome sequencing (WES) has been used as a single-step test in patients with hearing loss (Bademci et al., 2016; Zazo Seco et al., 2017; Sheppard et al., 2018; Sang et al., 2019). However, analyzing and interpreting WES data are usually laborious and time-consuming. A number of stepwise diagnostic approaches have been proposed (Baux et al., 2017; Guan et al., 2018; Li et al., 2019; Budde et al., 2020; Wang et al., 2021). Guan et al. (2018) used Sanger sequencing combined with targeted deletion analyses of *GJB2* and *STRC* and two mitochondrial genes, followed by WES analysis of deafness-related genes. This study revealed 11 diagnoses (33%) and 8 possible diagnoses (24.2%) in 33 NSHL patients. Wang et al. (2021) provided a two-tier strategy consisting of multiplex PCR plus next-generation sequencing (NGS) applied to detect *GJB2*, *SLC26A4*, and *MT-RNR1*, followed by WES analysis. A diagnosis was made in 64% (59/92) of the patients in this study, with 44 of these cases diagnosed by multiplex PCR combined with high-throughput sequencing. None of these studies examined exon 1 of the *GJB2* gene, which underestimated the diagnosis rate of the *GJB2* gene. The *SLC26A4* gene is the second leading cause of deafness in China. As Guan et al. (2018) did not include the *SLC26A4* gene in tier one, this strategy is not suitable for Chinese populations. Wang et al. (2021) did not evaluate the contribution of *STRC* CNVs to mild-to-moderate SNHL.

**FIGURE 1:**
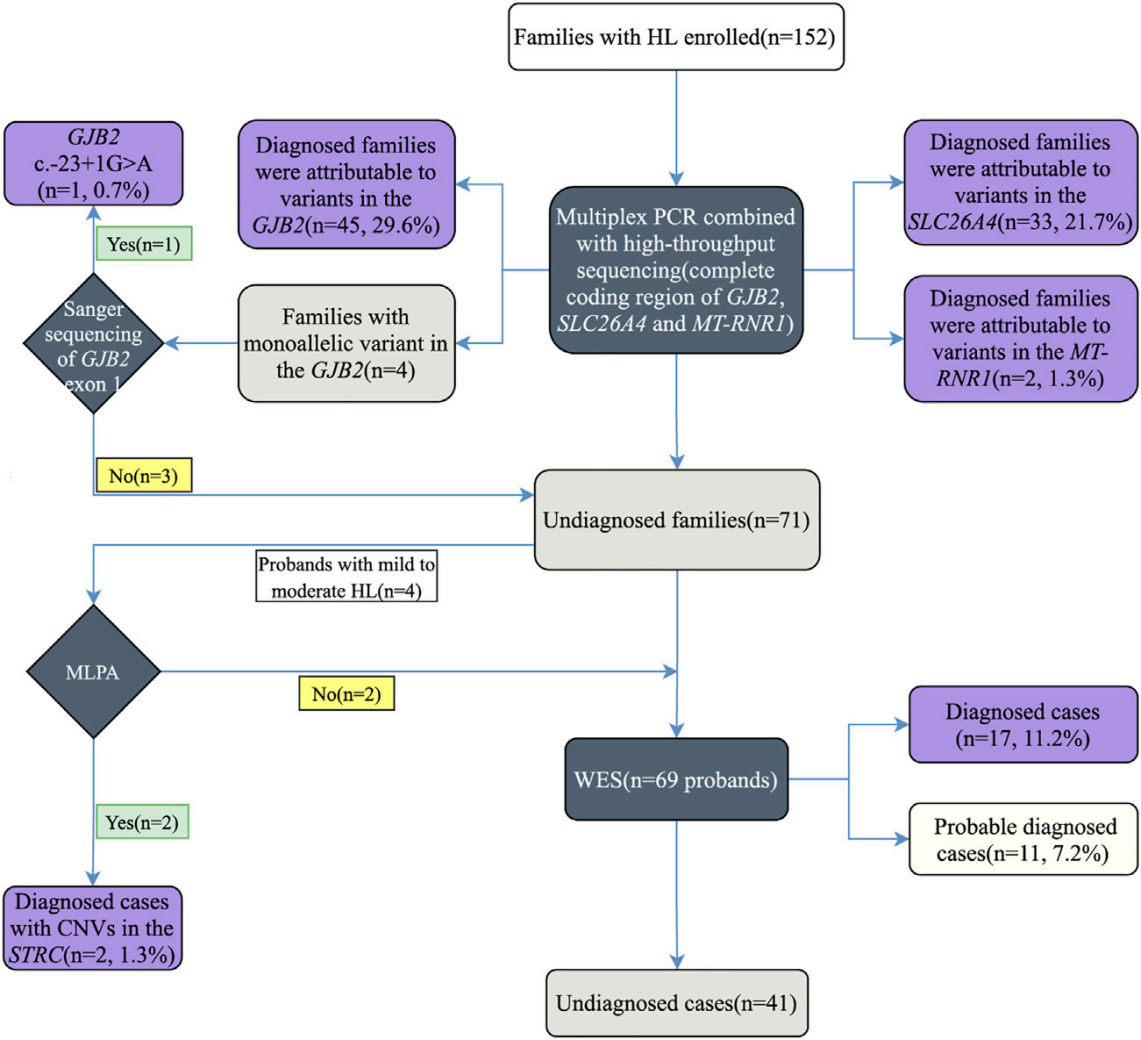
Study design and summary of stepwise genetic testing results.

Here, we propose a stepwise strategy including the detection of common deafness genes with multiplex PCR plus high-throughput sequencing, Sanger sequencing of *GJB2* non-coding exon 1, MLPA of the *STRC* gene, and WES (Figure 1). Our stepwise strategy is comprehensive and cost-effective in analyzing the contributions of different genetic factors to deafness. We achieved a diagnosis rate of 73% in 152 hearing loss families. With multiplex PCR plus high-throughput sequencing, we diagnosed 80 (52.6%) families. Sanger sequencing of *GJB2* exon 1 identified c.-23 + 1G>A variant in one patient. We detected two patients carrying homozygous deletion variants in the *STRC* gene by MLPA. Among the remaining undiagnosed patients, we obtained 17 diagnoses and 11 probable diagnoses by WES. The result can provide accurate and comprehensive genetic counseling for deafness families, and provide a strong foundation for prevention and control of birth defects of deafness in the family. In summary, this study improved the diagnostic yield in a cohort of hearing loss families, and confirmed the advantages of a stepwise strategy for molecular diagnosis of hearing loss.

## Materials and methods

### Subjects

Our cohort consisted of 152 hearing loss families from a special education school in Zhengzhou, Henan Province, China. The level of hearing loss was classified into four tiers in terms of pure tone average (averaged over 0.5, 1.0, 2.0, and 4.0 kHz): mild (21–40 dB nHL), moderate (41–70 dB nHL), severe (71–90 dB nHL), and profound (>91 dB nHL). This study complied with the World Medical Association Declaration of Helsinki and was approved by the Medical Ethics Committee of the Second Affiliated Hospital of Zhengzhou University (approval number: 2018008). Each participant provided signed informed consent (for probands under 18 years old, written informed consent was provided by their guardians) before participate in the present study.

### Study design

All family members of the cohort were first tested for pathogenic variants in *GJB2*, *SLC26A4*, and *MT-RNR1* genes (Figure 1). Sanger sequencing of *GJB2* exon 1 was then carried out in undiagnosed families carrying *GJB2* monoallelic variants. In addition, the *STRC* gene was tested in probands with mild-to-moderate hearing loss by MLPA. Finally, undiagnosed probands were referred for WES. Due to the variable expressivity and penetrance of c.109G>A in *GJB2* (Shen et al., 2019), probands diagnosed with this variant (homozygous or compound heterozygous with other *GJB2* variants) were also referred for WES to explore other potential molecular etiologies.

### Multiplex PCR, high-throughput sequencing, and bioinformatics analysis

Genomic DNA was extracted from 2 ml of peripheral blood using a DNA extraction kit (GenMagBio, Amherst, NY, United States). NanoDrop One (Thermo Fisher Scientific, Waltham, MA, United States) was used to measure DNA concentration and purity. A multiplex PCR kit was designed to cover the entire coding region of *GJB2*, *SLC26A4*, and *MT-RNR1*, as an updated version of the kit developed in a previous study (Tian et al., 2021). Multiplex PCR assay does not include detection of the *GBJ2* exon 1. The resulting libraries were sequenced on an Illumina MiniSeq sequencer (Illumina, San Diego, CA, United States) in 150-bp paired-end mode. Bioinformatics analysis was performed in the bcbio-nextgen framework (https://github.com/bcbio/bcbio-nextgen). Reads trimming, genome aligning, variant annotation, filtering, and interpretation were carried out as described previously (Tian et al., 2021).

### Sanger sequencing of *GJB2* exon 1

*GJB2* exon 1, its c.-23 + 1G>A variant and variants in the promoter region were amplified with the primers (Supplementary Table S1) in four families with a monoallelic variant in the coding region of *GJB2*.

### Multiplex ligation-dependent probe amplification analysis of the *STRC* gene

Among probands undiagnosed using multiplex PCR combined with high-throughput sequencing, we screened all patients with mild-to-moderate hearing loss for MLPA. An MLPA probemix kit (Catalogue numbers: P461-A1-050R, MRC-Holland, Amsterdam, the Netherlands) was used to detect CNVs of *STRC*, as suggested by the manufacturer. Amplification products were run on an Applied Biosystems™ SeqStudio™ Genetic Analyzer (Thermo Fisher Scientific), and the results were analyzed using GeneMarker 1.91 software (SoftGenetics, State College, PA, United States).

### Whole-exome sequencing and bioinformatics analysis

WES was applied to identify the potential genetic causes for probands undiagnosed in the steps described above. Standard WES-based genetic testing, including sample preparation and quantification, library construction, hybrid sequence capture, sequencing, and data analyses, was performed as described previously (Pan et al., 2020; Zhang et al., 2021). Copy number analysis was performed from NGS data using DECoN (Fowler et al., 2016) with the alignment BAM files from the same enrichment panel and sequencing run.

### Sanger validation and co-segregation analysis

We performed PCR amplification and Sanger sequencing to confirm candidate variants detected by WES and conduct co-segregation analyses in family members. The specific primers (Supplementary Table S1) were designed using NCBI Primer-BLAST and synthesized by Sunya Biotech Co., Ltd. (Zhengzhou, China). Sequencing was performed using a SeqStudio Genetic Analyzer (Applied Biosystems, Foster City, CA, United States) after PCR product purification, and the results were visualized with Chromas software.

### Variant interpretation and the definition of molecular diagnosis

Variant interpretation was performed according to the guidelines of the American College of Medical Genetics and Genomics (ACMG) (Richards et al., 2015) and the ClinGen Hearing Loss Expert Group’s recommendations (Oza et al., 2018). According to the GenCC database (https://search.thegencc.org/), the candidate gene was included in the results only when there was a definitive, strong, or moderate relationship between the gene and deafness. Patients were categorized as “diagnosed” if they were homozygous or compound heterozygous for a pathogenic/likely pathogenic variant(s) in a recessively inherited gene or heterozygous for a pathogenic/likely pathogenic variant in a dominantly inherited gene. In addition, patients with a pathogenic/likely pathogenic variant plus a rare variant of uncertain significance (VUS) in a recessively inherited gene, or two rare VUS in the *CDH23* or *MYO15A* gene, were considered as “probably diagnosed.” When there were two VUS in the candidate pathogenic gene of the patient, and the clinical phenotype caused by the gene variant was consistent with the clinical phenotype of the patient, it was also considered as “probably diagnosed.” Patients with one pathogenic/likely pathogenic variant in a recessively inherited gene were considered as “undiagnosed.”

## Results

### Clinical characteristics of the probands

We recruited 152 hearing loss families from a special education school in Zhengzhou City, Henan Province, China. The probands included 100 (65.8%) males and 52 (34.2%) females; 131 (86.2%) patients had prelingual hearing loss and 21 (13.8%) patients were postlingual; 139 (91.5%) patients had severe-to-profound deafness, 9 (5.9%) patients had mild-to-moderate hearing loss, and 4 (2.6%) patients had no records; 128 (84.2%) patients showed stable hearing loss, 21 (13.8%) patients were progressive, and 3 (2%) patients had no records. A few patients were suspected to have SHL (2%) before genetic testing, and the majority (98%) were found to have NSHL (Table 1).

**TABLE 1:** Clinical features of the probands.

Characteristic	Number of probands (%)
All	152(100.0)
Gender	
Male	100(65.8)
Female	52(34.2)
Onset age (years)	
Prelingual (0–2)	131(86.2)
Postlingual (>2)	21(13.8)
Degree of HL	
Profound	96(63.2)
Severe	43(28.3)
Moderate	5(3.3)
Mild	4(2.6)
No record	4(2.6)
Stability	
Stable	128(84.2)
Progressive	21(13.8)
No record	3(2.0)
Characteristics of HL	
Non-syndromic	149(98.0)
Syndromic	3(2.0)

### Multiplex PCR diagnoses in 80 hearing loss families

**FIGURE 2:**
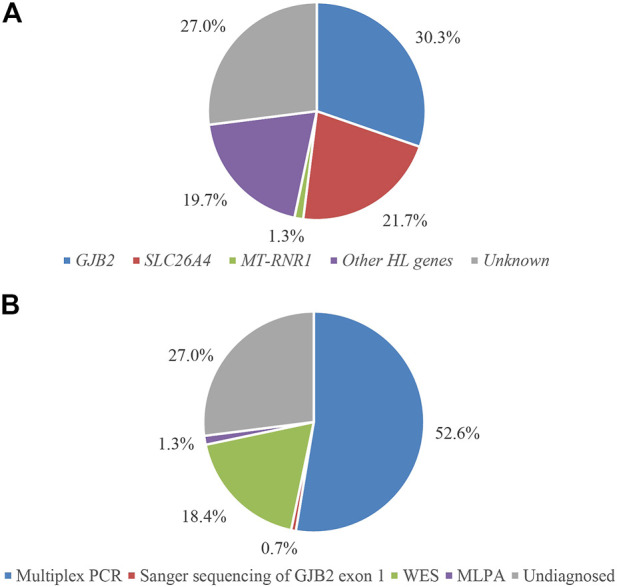
Molecular diagnostic results of 152 families classified according to genes **(A)** and detection methods **(B)**.

All family members in 152 families were tested for mutations in the common deafness genes by multiplex PCR plus high-throughput sequencing. The average sequencing depth of target regions was 500×, with 100% of target regions having coverage greater than 100×. The diagnosis rate of multiplex PCR combined with high-throughput sequencing was 52.6% (80/152), of which *GJB2* accounted for 29.6% (45/152), *SLC26A4* accounted for 21.7% (33/152), and *MT-RNR1* accounted for 1.3% (2/152) (Figures 2A,B). The genotypes of the 80 patients who tested positive are listed in Table 2. The variant c.235del was the most prevalent in the *GJB2* gene, including 19 patients who were homozygous and 22 patients who were compound heterozygous. The variant c.919-2A>G was the most prevalent in the *SLC26A4* gene, including 10 patients who were homozygous and 16 patients who were compound heterozygous. The patients whose pathogenic gene was *MT-RNR1* carried a homoplasmic m.1555A>G variant. One patient carried *SLC26A4* c.1124A>G and c.1409G>A variants both of which came from the mother, and there was no enlarged vestibular aqueduct (EVA) in clinical diagnosis. In addition, four patients have a single heterozygous variant of *GJB2* (Supplementary Table S2). Therefore, Sanger sequencing of *GJB2* exon 1 was carried in these four families. We detected one patient carrying the c.-23 + 1G>A variant, forming a compound heterozygous variant with c.235del (Table 3). The remaining three patients were further examined by WES.

**TABLE 2:** Genotypes of 80 patients detected by multiplex PCR sequencing. Hom, homozygous; Het, heterozygous; Homo, homoplasmy; AR, autosomal recessive; Mi, mitochondrial; P, pathogenic; LP likely pathogenic.

No	Gene, Transcript	Variant 1	Zygosity	ACMG classification	Variant 2	Zygosity	ACMG classification	Inheritance	Number of patients
1	*GJB2*, NM_004004.6	c.235del, p.(Leu79CysfsTer3)	Hom	P	—	—	—	AR	19
2		c.235del, p.(Leu79CysfsTer3)	Het	P	c.299_300del, p.(His100ArgfsTer14)	Het	P	AR	9
3		c.235del, p.(Leu79CysfsTer3)	Het	P	c.176_191del, p.(Gly59AlafsTer18)	Het	P	AR	4
4		c.235del, p.(Leu79CysfsTer3)	Het	P	c.109G>A, p.(Val37Ile)	Het	P	AR	2
5		c.299_300del, p.(His100ArgfsTer14)	Het	P	c.257C>G, p.(Thr86Arg)	Het	P	AR	2
6		c.235del, p.(Leu79CysfsTer3)	Het	P	c.427C>T, p.(Arg143Trp)	Het	P	AR	2
7		c.299_300del, p.(His100ArgfsTer14)	Hom	P	—	—	—	AR	1
8		c.235del, p.(Leu79CysfsTer3)	Het	P	c.257C>G, p.(Thr86Arg)	Het	P	AR	1
9		c.235del, p.(Leu79CysfsTer3)	Het	P	c.139G>T, p.(Glu47Ter)	Het	P	AR	1
10		c.235del, p.(Leu79CysfsTer3)	Het	P	c.127G>C, p.(Val43Leu)	Het	LP	AR	1
11		c.235del, p.(Leu79CysfsTer3)	Het	P	c.35del, p.(Gly12ValfsTer2)	Het	P	AR	1
12		c.508_511dup, p.(Ala171GlufsTer40)	Het	P	c.9G>A, p.(Trp3Ter)	Het	P	AR	1
13		c.235del, p.(Leu79CysfsTer3)	Het	P	c.508_511dup, p.(Ala171GlufsTer40)	Het	P	AR	1
14	*SLC26A4*, NM_000441.2	c.919-2A>G, -	Hom	P	—	—	—	AR	10
15		c.919-2A>G, -	Het	P	c.2168A>G, p.(His723Arg)	Het	P	AR	3
16		c.919-2A>G, -	Het	P	c.1174A>T, p.(Asn392Tyr)	Het	P	AR	3
17		c.2168A>G, p.(His723Arg)	Het	P	c.1975G>C, p.(Val659Leu)	Het	P	AR	2
18		c.919-2A>G, -	Het	P	c.2027T>A, p.(Leu676Gln)	Het	P	AR	2
19		c.919-2A>G, -	Het	P	c.1975G>C, p.(Val659Leu)	Het	P	AR	1
20		c.916dup, p.(Val306GlyfsTer24)	Het	P	c.1149 + 1G>A, -	Het	P	AR	1
21		c.2168A>G, p.(His723Arg)	Het	P	c.1707 + 5G>A, -	Het	P	AR	1
22		c.919-2A>G, -	Het	P	c.1229C>T, p.(Thr410Met)	Het	P	AR	1
23		c.919-2A>G, -	Het	P	c.1707 + 5G>A, -	Het	P	AR	1
24		c.919-2A>G, -	Het	P	c.1105A>G, p.(Lys369Glu)	Het	P	AR	1
25		c.2168A>G, p.(His723Arg)	Het	P	c.1327G>C, p.(Glu443Gln)	Het	P	AR	1
26		c.1174A>T, p.(Asn392Tyr)	Het	P	c.1586T>G, p.(Ile529Ser)	Het	P	AR	1
27		c.919-2A>G, -	Het	P	c.1001 + 2T>A, -	Het	P	AR	1
28		c.919-2A>G, -	Het	P	c.203T>C, p.(Leu68Pro)	Het	LP	AR	1
29		c.919-2A>G, -	Het	P	c.1149 + 1G>A, -	Het	P	AR	1
30		c.1226G>A, p.(Arg409His)	Het	P	c.1520del, p.(Leu507Ter)	Het	P	AR	1
31	*SLC26A4*, NM_000441.2	c.919-2A>G, -	Het	P	c.2168A>G, p.(His723Arg)	Het	P	AR	1
	*GJB2*, NM_004004.6	c.109G>A, p.(Val37Ile)	Hom	P	—	—	—	AR	
32	*MT-RNR1*	m.1555A>G	Homo	P	—	—	—	Mi	2

**TABLE 3:** *GJB2* c.-23 + 1G>A varaint in one patient (single heterozygous).

*GJB2* exon 2					Exon 1 or splice site			
Patient id	Gene, Transcript	Variant 1	Zygosity	ACMG classification	Variant 2	Zygosity	ACMG classification	Inheritance
3312201	*GJB2*, NM_004004.6	c.235del, p.(Leu79CysfsTer3)	Het	P	c.-23 + 1G>A, -	Het	P	AR
3312209		c.235del, p.(Leu79CysfsTer3)	Het	P	—	—	—	—
3312306		c.235del, p.(Leu79CysfsTer3)	Het	P	—	—	—	—
3312282		c.109G>A, p.(Val37Ile)	Het	P	—	—	—	—

### MLPA analysis of *STRC* gene diagnoses of two mild-to-moderate NSHL patients

Of the 71 undiagnosed patients, four had mild-to-moderate NSHL, and were examined by MLPA. We detected two patients carrying homozygous deletion variants in exons 19 and 23–25 of the *STRC* gene (Table 4), and the homozygous form may be the primary cause of hearing loss. The female patient was diagnosed with homozygous continuous gene deletion of *STRC* and *CATSPER2*. However, if the proband were male, he may have suffered from deafness-infertility syndrome (MIM611102) (Avidan et al., 2003; Zhang et al., 2007).

**TABLE 4:** Genotypic spectrum of four mild-to-moderate NSHL patients.

Large CNV detection by MLPA							
Patient id	Gender	Variant	Zygosity	Inheritance	Variant	Zygosity	Inheritance
		*STRC*, NM_153700.2			*CATSPER2*, NM_172095.4		
3310326	Female	Exons 19 and 23–25 deletion	Hom	AR	Exons 1, 2, 4, 7, and 12 deletion	Hom	AR
3310270	Male	Exons 19 and 23–25 deletion	Hom	AR	Exons 1, 2, 4, 7, and 12 deletion	Het	AR
3312308	Female	—	—	—	—	—	—
3312236	Male	—	—	—	—	—	—

### WES diagnoses/probable diagnoses in 28 cases

Sixty-nine undiagnosed patients were referred for WES, resulting in 17 diagnoses and 11 probable diagnoses (Supplementary Table S2). The diagnosis rate with additional contribution from WES was 18.4% (28/152). In the 28 cases, a total of 46 different variants were detected in 15 known deafness genes, of which 21 (21/46, 45.7%) were novel from *TRIOBP*, *MPZL2*, *MYO15A*, *PCDH15*, *LOXHD1*, *POU3F4*, *CDH23*, *COL11A2*, and *HARS2* genes. All detected variants fulfilled the requirement for phenotype–genotype co-segregation. The genes with the largest proportion of patients detected were *CHD23* (5/28, 17.9%) and *MYO15A* (6/28, 21.4%). One patient 3312236) with mild-to-moderate NSHL was negative on the *STRC* MLPA test, and the pathogenic gene was identified as *MPZL2* by WES. This was the first reported case of mild-to-moderate hearing loss caused by *MPZL2* variants in China.

In addition, due to the variable expressivity and incomplete penetrance of *GJB2* c.109G>A (Shen et al., 2019), two compound heterozygous patients for *GJB2* c.109G>A identified by multiplex PCR were also referred for WES to exclude other potential molecular etiologies. No other causally associated hearing loss variants were identified in these two patients by these analyses. Therefore, we included these two patients in the group diagnosed by multiplex PCR combined with high-throughput sequencing. The molecular diagnosis results of 152 hearing loss families are shown in Figure 2. The results of Sanger validation of families are shown in Supplementary Figure S1.

### WES combined with deep clinical phenotyping revealed 11 patients with syndromic hearing loss

Combining WES and deep clinical phenotyping, we diagnosed 11 patients with SHL. Four had Waardenburg syndrome (WS)-II type A and type E, caused by *MITF* and *SOX10* gene variants, respectively. Before genetic testing, patients 3312301 and 3312311 were primarily diagnosed with WS with congenital bilateral profound SNHL and blue iris. In contrast, patients 3312176 and 3312249 were diagnosed with NSHL. After genetic testing, we detected a *de novo* variant in the *SOX10* gene in patient 3312301 and a *de novo* variant of the *MITF* gene in patient 3312311. Their clinical phenotypes were consistent with the genotypes and could be identified as WS-II. We found that *MITF* was a candidate pathogenic gene for patients 3312176 and 3312249. Therefore, we revisited the two families for further clinical phenotypic examination. It is worth noting that patient 3312176 had profound bilateral deafness and freckles caused by NM_198159.3 (*MITF*): c.1212G>A, but there was no other clinical phenotype associated with WS. The variant came from the father, and the proband’s sister also had this variant, but only had freckles and had normal hearing. A similar situation occurred in patient 3312249 (*MITF* c.1021C>T). The proband had clinical phenotypes of deafness and freckles but no other classic phenotype of WS. The variant came from the mother, who has normal hearing and only a few freckles. Therefore, we confirmed that the patients in these two families had WS-II.

Before genetic testing, patients 3312205 and 3312291 were primarily diagnosed with NSHL, and patient 3312343 was included as “syndromic” because of her small stature and progeroid appearance with hearing loss. After a genetic diagnosis, we found two *de novo* variants of *PTPN11* in patients 3312205 and 3312291, and one *de novo* variant of the *POLD1* gene in patient 3312343, which led to diagnoses of Noonan syndrome (MIM163950) and MDPL syndrome (mandibular hypoplasia, deafness, progeroid features, and lipodystrophy syndrome, MIM615381), respectively. The case of patient 3312343 was described in detail in another article. For patient 3312205, we conducted an in-depth clinical examination and the results revealed congenital heart disease and atrial septal defect, which could be identified as Noonan syndrome. We also informed the parents of patient 3312291 of the genetic testing results and suggested that relevant clinical examinations should be carried out as soon as possible to determine the etiology of the proband and prevent more serious disease consequences.

In addition, we also found that the pathogenic gene of an early childhood patient, 3312198, was *USH2A*. Although the patient was primarily diagnosed with NSHL before genetic testing, the results showed that he had Usher syndrome (type 2A) (MIM276901). We found two frameshift variants of the *PCDH15* gene in an early childhood patient, 3312279. Before genetic testing, the patient was primarily diagnosed with NSHL. However, the results showed that she was likely to have Usher syndrome. We recommended that the guardian accompany the patient regularly to check her motor development and visual acuity, and follow the doctor’s treatment advice. Patient 3312229 was identified as a compound heterozygote composed of splicing and missense variants of the *CDH23* gene. Although the patient 3312229 was more likely to have Usher syndrome, we cannot exclude the possibility of non-syndromic hearing loss. The patient was only in early childhood, and there was no syndrome-related phenotype, such as retinitis pigmentosa, which may occur at a relatively late age.

We detected two missense variants of *HARS2* in an early childhood boy, patient 3312255, who was primarily diagnosed with NSHL before genetic testing. Variants in this gene lead to a diagnosis of Perrault syndrome 2 (MIM614926). Females with this disease will have clinical phenotypes, such as ovarian dysgenesis and infertility, while affected males have normal pubertal development and fertility.

Therefore, WES combined with deep phenotyping improved diagnostic yield, and comprehensive and complete phenotypic information is indispensable for genetic analysis of hearing loss.

## Discussion

This study proposed a stepwise strategy of deafness genes with multiplex PCR combined with high-throughput sequencing, Sanger sequencing, MLPA, and WES to explore molecular diagnoses of 152 Chinese hearing loss families, achieving a 73% (111/152) diagnostic yield. Our study had a higher diagnosis rate than the stepwise detection strategies reported in previous studies (48%–64%) (Baux et al., 2017; Wang et al., 2021).

Our study used multiplex PCR plus NGS to detect mutations in the *GJB2*, *SLC26A4,* and *MT-RNR1* genes with a diagnosis rate of 52.6%, providing rapid molecular diagnosis and saving the cost of WES. This is because there are hotspot genes and variants in the Chinese hearing loss population, such as *GJB2* c.235del, *SLC26A4* c.919-2A>G, and *MT-RNR1* m.1555A>G. Compared with the diagnostic strategy of analyzing only the *GJB2* gene in the first step (Baux et al., 2017; Guan et al., 2018), our multiplex PCR detection strategy is more effective and economical for the Chinese hearing loss population. In addition, for Chinese patients with hearing loss, most detection strategies reported to date may underestimate the diagnosis rate of the *GJB2* gene because they did not examine mutations in exon 1 (Sakuma et al., 2016; Guan et al., 2018; Wang et al., 2021). The *GJB2* c.-23 + 1G>A variant accounts for 0.2%–1.89% of hearing loss patients in China (Yuan et al., 2010; Yu et al., 2020).

It is reported that *MT-RNR1* m.1555A>G variant may lead to low or non-penetrance hearing loss (Li et al., 2004). In the absence of aminoglycosides, the m.1555A>G variant produces a clinical phenotype that ranges from severe congenital deafness, to moderate progressive hearing loss of later onset, to completely normal hearing (Prezant et al., 1993; Estivill et al., 1998). If there are patients with deafness who have m.1555A>G variant and no history of aminoglycosides exposure, we suggest that WES should be used to explore other potential molecular etiologies.

If there are mild-to-moderate hearing loss patients in the cohort, then it would also be necessary to include MLPA detection of the *STRC* gene in the stepwise strategy. Although there have been few studies on hearing loss caused by the *STRC* gene in China, Yokota et al. (2019) and Kim et al. (2020) emphasized that the *STRC* gene is the main cause of mild-to-moderate hearing loss in the East Asian population. However, Wang et al. (2021) did not analyze *STRC* CNVs in their detection strategy. Our study also confirmed the above inference as two of the four patients with mild-to-moderate hearing loss in the cohort were diagnosed by MLPA testing.

Combining WES and deep clinical phenotyping, we revealed a number of cases of SHL. WS has a high degree of genetic heterogeneity and highly variable phenotype expressivity (Newton, 1990; Read and Newton, 1997; Song et al., 2016). We identified two families with WS caused by *MITF* gene variants, and their members had different degrees of phenotype expressivity. *PCDH15* and *CDH23* are the pathogenic genes for Usher syndrome. It was reported that missense variants of the *PCDH15* gene led to NSHL, while other variants (frameshift, nonsense, splicing, and large fragment deletion) caused Usher syndrome (type 1F) (MIM602083) (Ahmed et al., 2008). Li et al. (2020) reported a family with Usher syndrome type 1 caused by two frameshift variants in the *PCDH15* gene. This suggested that patient 3312279 was likely to have Usher syndrome. Similarly, it was reported that nonsense variants, splicing variants, and frameshift variants in the *CDH23* gene led to Usher syndrome (type 1D) (MIM601067), while missense variants led to DFNB12 (Bork et al., 2001; Astuto et al., 2002). We identified a splicing variant and a missense variant of the *CDH23* gene in patient 3312229. Our previous study confirmed that compound heterozygosity of *CDH23* splicing and missense variants led to Usher syndrome (Chen et al., 2020). Therefore, the patient was more likely to have Usher syndrome. These cases highlight the advantages of WES in diagnosing hearing loss-related syndromes. This allows the early recognition of the syndrome, especially Usher syndrome, in patients and suggests the effect of cochlear implantation and reduces the risk of disease recurrence in families.

We identified 11 probable diagnoses in probands by WES, of which five were attributed to variants in the *CHD23* gene and three to variants in the *MYO15A* gene. This is because the main type of variation detected in these two genes is missense variation and the functional effects of variants cannot be determined. Most missense variants can only be interpreted as VUS by following the ACMG guidelines. Experimental validation of the possible pathogenic effects by *in vivo* or *in vitro* functional studies is an accurate method of determining the molecular basis of disease caused by missense variants (George Priya Doss et al., 2013). Deep mutational scanning may also be useful (Fowler and Fields, 2014). In addition, practical bioinformatics tools can be used to predict the functional effects of missense variants (Ng and Henikoff, 2006). Many disease-related missense variants have been evaluated by *in silico* methods, and have helped to reveal protein structure–function relationships and disease genotype–phenotype correlations (Thusberg and Vihinen, 2006; Doss and Sethumadhavan, 2009; Forman et al., 2009).

In this study, up to 111 families with deafness can be diagnosed, but effective treatment is still lacking. Although current treatments, such as hearing aids and cochlear implants, can rehabilitate hearing in some patients, these approaches are limited by their sensitivity and perception of natural sounds in noisy environments (Lesica, 2018; Tan et al., 2019). Hearing loss is mainly caused by the irreversible loss of cochlear hair cells and auditory neurons. Thus, an effective way to treat hearing loss is to regenerate or repair damaged cells in the inner ear. During the past decade, gene therapy and neural stem cell therapy have emerged as promising treatment strategies for hearing loss (He et al., 2021; Nourbakhsh et al., 2021; Qi et al., 2022). One major challenge of gene therapy is effectively delivering genes to target cells, and at present, the delivery vectors contain two types: viral and non-viral. Among viral vectors, the Adeno-associated virus (AAV) vector is suitable for the treatment of hearing loss with a number of AAV serotypes have been developed, such as AAV1(Akil et al., 2012), AAV-ie (Tan et al., 2019; Tao et al., 2022), AAV9-PHP.B (Taiber et al., 2021), *etc.* In addition to gene therapy, using stem cells to induce differentiation to regenerate or repair damaged cells in the inner ear is regarded as the most feasible treatment (He et al., 2021). There are usually two routes of research: the transplantation of exogenous stem cells and their subsequent differentiation in the damaged tissue and the genetic modification of endogenous cells to promote their transdifferentiation potential (de Felipe et al., 2011; Duran-Alonso, 2020). We predict that, with the application of biomaterials in stem cell therapy (Xia et al., 2022), pathogenesis-based gene therapy and stem cell therapy will be ideal for human hereditary deafness.

Forty-one patients in this study were still undiagnosed, among whom two negative patients had a single heterozygous variant of *GJB2*, and one patient carried *SLC26A4* (c.1124A>G and c.1409G>A) variants with a single maternal haplotype, and there was no EVA in clinical diagnosis. No pathogenic variants were found in the remaining patients. There were two families in the cohort, each with two patients with prelingual deafness suggesting a monogenic trait, and the molecular etiology was not identified by WES. We may conduct further research in the future. The hearing loss in some of the patients may not have had a genetic etiology, e.g., patient 3312294 was suspected to have post-traumatic hearing loss based on the clinical phenotypic survey. In addition, undiagnosed patients may have hearing loss caused by new deafness genes that have not been identified previously or variants that could not be detected by the methods used in this study. In the next step, we will perform further analyses of these patients, such as WGS and third-generation sequencing.

In conclusion, this study improved the diagnostic yield, revealed rich phenotypic differences, and confirmed the advantages of a stepwise strategy in the molecular diagnosis of hearing loss families.

## Data Availability

The raw sequencing data supporting this article cannot be placed in public repository due to national legislation/guidelines, specifically the Regulation of the People's Republic of China on the Administration of Human Genetic Resources (http://www.gov.cn/zhengce/content/2019-06/10/content_5398829.htm, http://english.www.gov.cn/policies/latest_releases/2019/06/10/content_281476708945462.htm) and institutional guidelines by the special education school. The exome sequencing data for undiagnosed patients were deposited in the National Supercomputing Center in Zhengzhou. Please email nscc@zzu.edu.cn for detailed application guidance only for academic use.
