# Supplementary material for: Increased diagnostic yield in a cohort of hearing loss families using a comprehensive stepwise strategy of molecular testing
Source: Front Genet. 2022 Dec 7;13:1057293. doi: 10.3389/fgene.2022.1057293 (PMC9768221; doi:10.3389/fgene.2022.1057293)

Supplementary Figure

1 Supplementary Figure S1

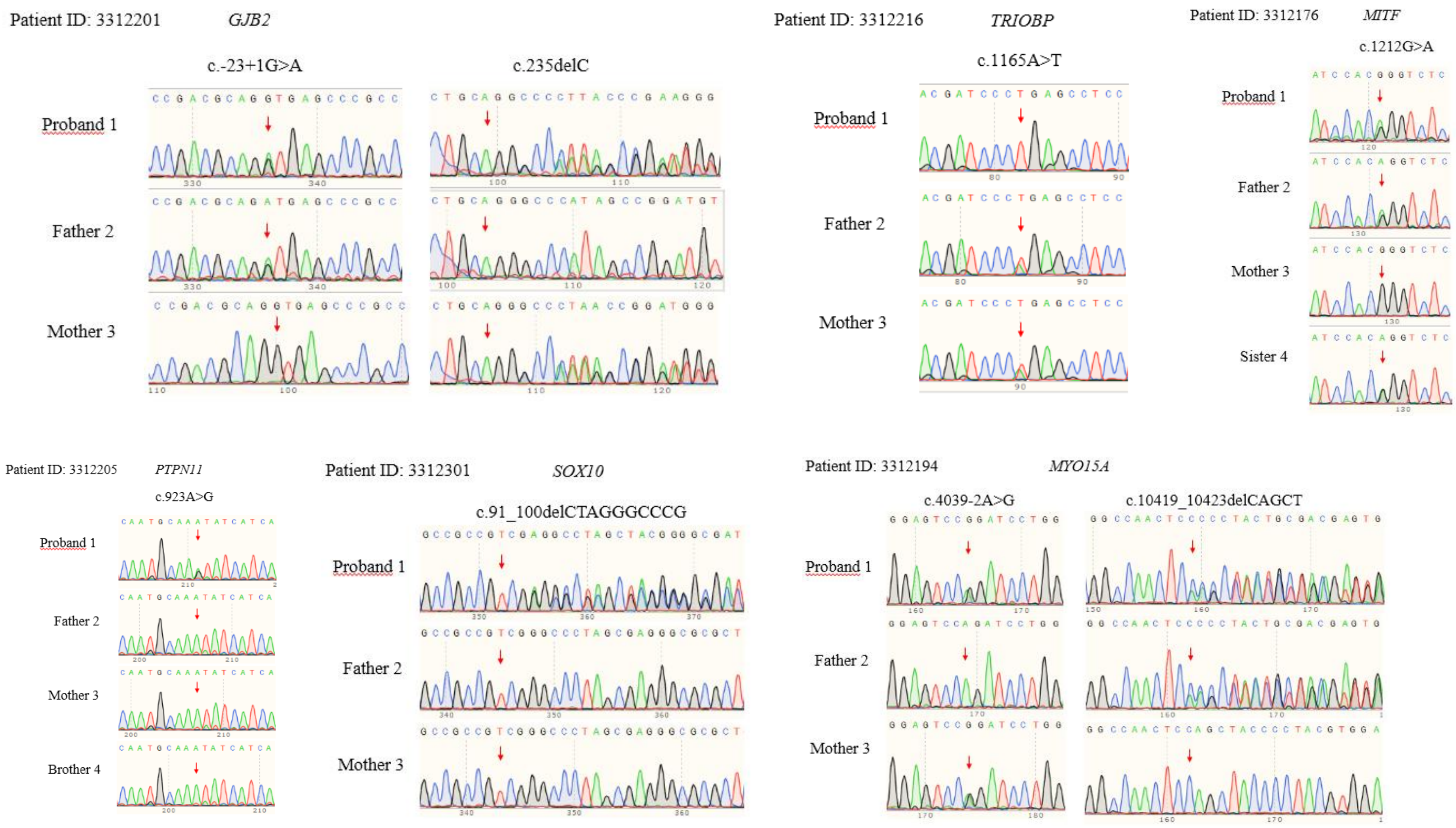

Patient ID: 3312198 *USH2A*

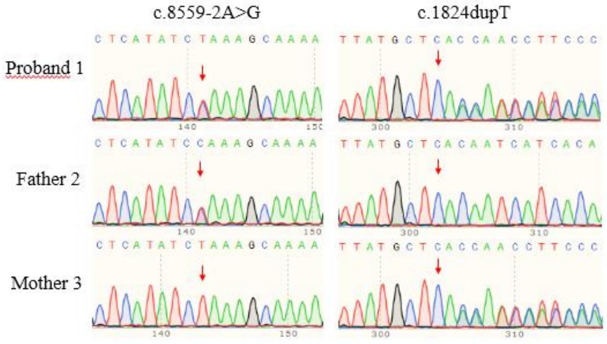

Patient ID: 3312236 *MPZL2*

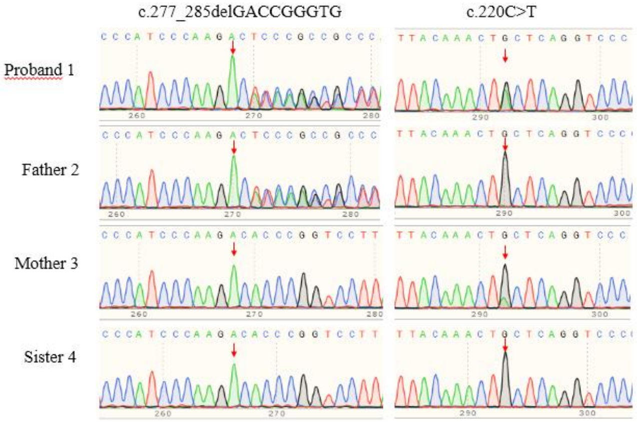

Patient ID: 3312291 *PTPN11*

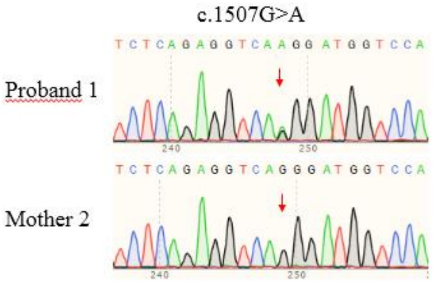

Patient ID: 3312313 *TMPRSS3*

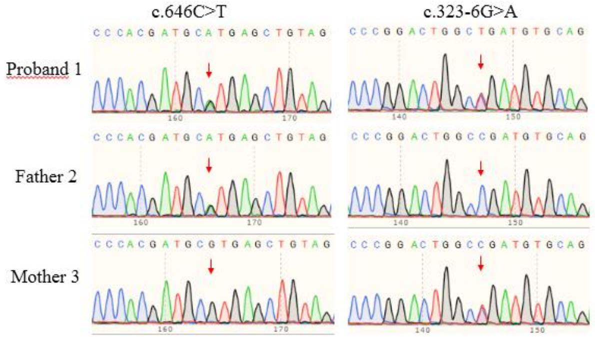

Patient ID: 3312279 *PCDH15*

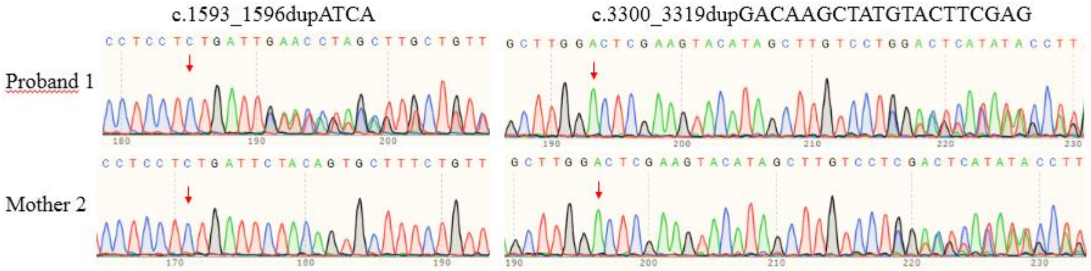

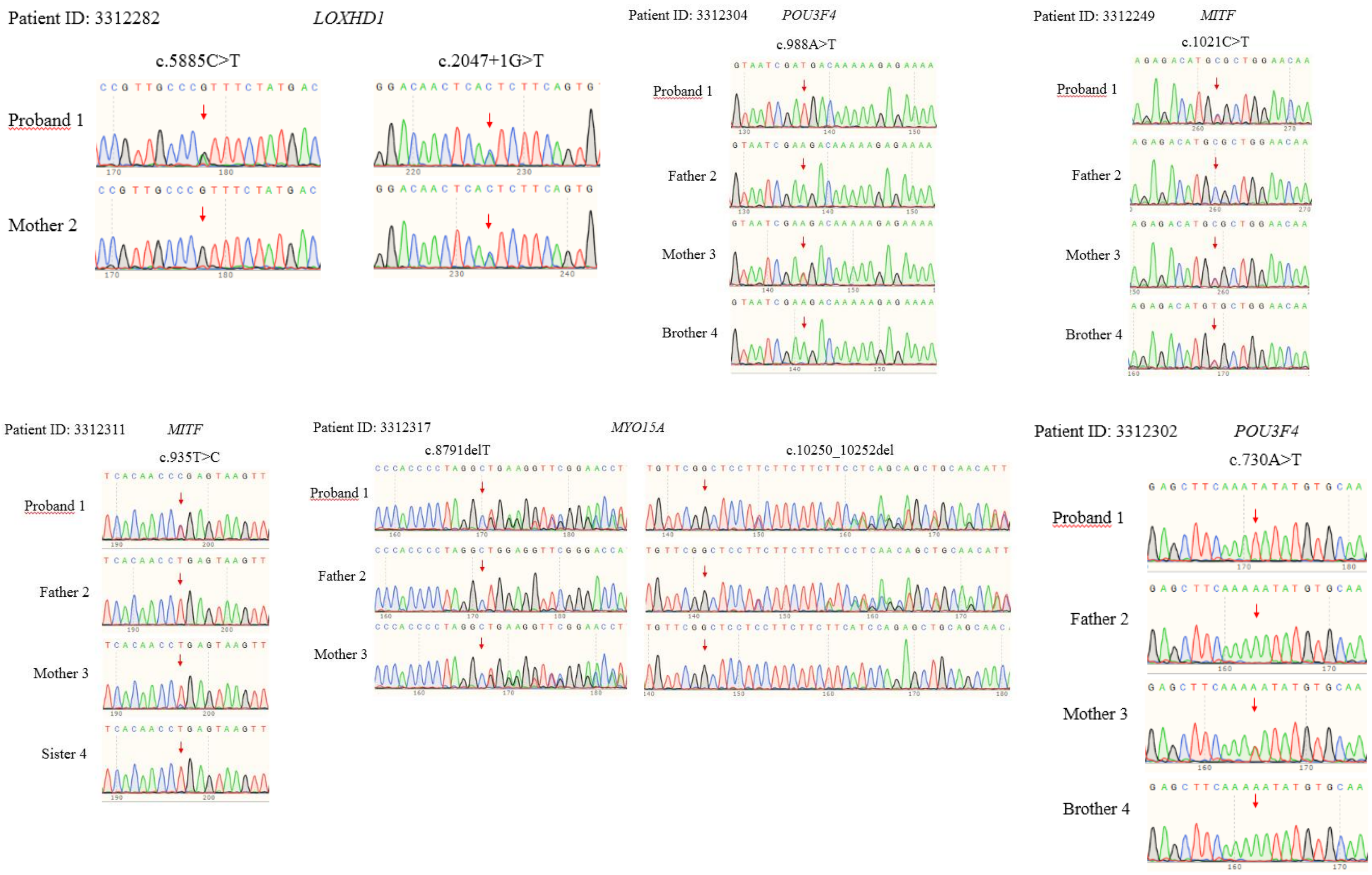
Patient ID: 3312282
LOXHD1
c.5885C>T
c.2047+1G>T
Proband 1
Mother 2
Patient ID: 3312304
POU3F4
c.988A>T
Proband 1
Father 2
Mother 3
Brother 4
Patient ID: 3312249
MITF
c.1021C>T
Proband 1
Father 2
Mother 3
Brother 4
Patient ID: 3312311
MITF
c.935T>C
Proband 1
Father 2
Mother 3
Sister 4
Patient ID: 3312317
MYO15A
c.8791delT
c.10250_10252del
Proband 1
Father 2
Mother 3
Patient ID: 3312302
POU3F4
c.730A>T
Proband 1
Father 2
Mother 3
Brother 4

Patient ID: 3312298 *MYO15A*

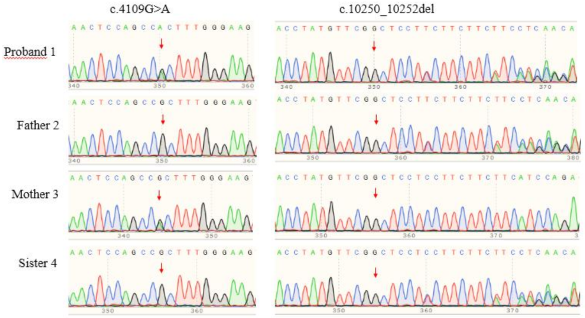

Patient ID: 3312167 *CDH23*

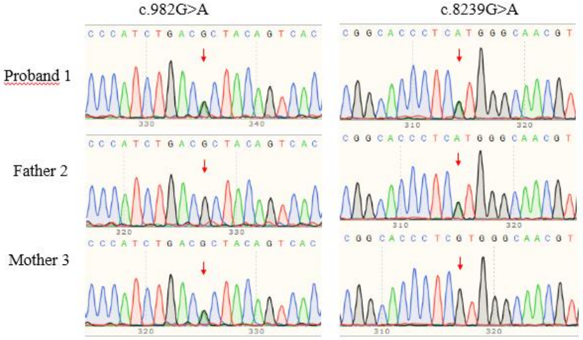

Patient ID: 3312225 *MYO15A*

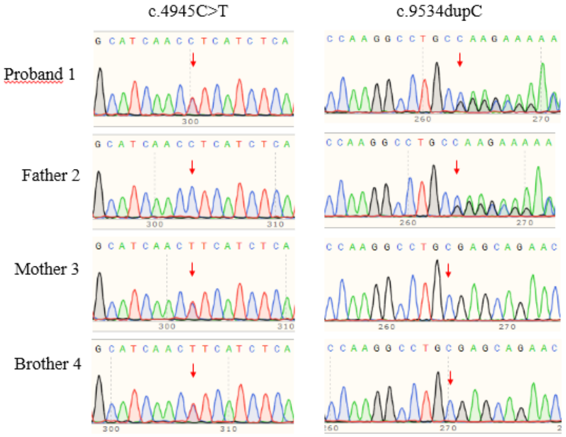

Patient ID: 3312234 *CDH23*

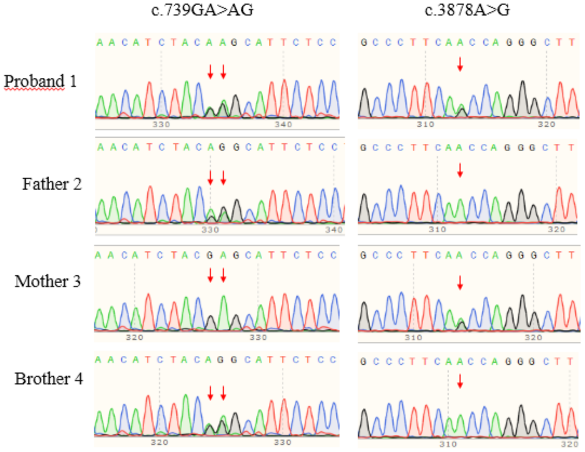

Patient ID: 3312358 *COL11A2*

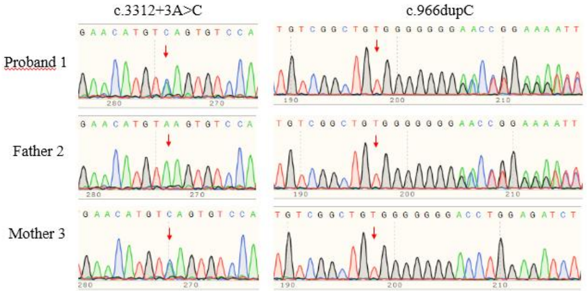

Patient ID: 3312206 *CDH23*

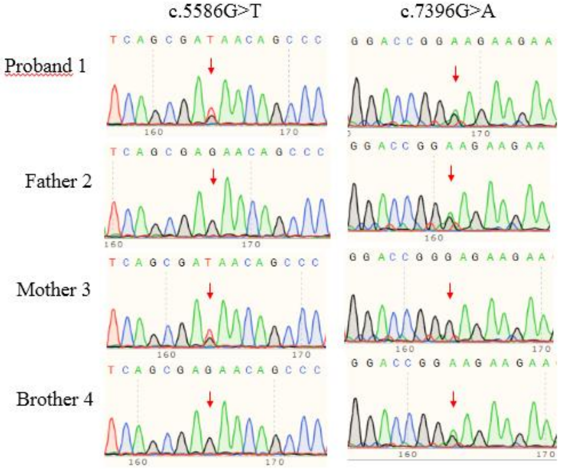

Patient ID: 3312255 *HARS2*

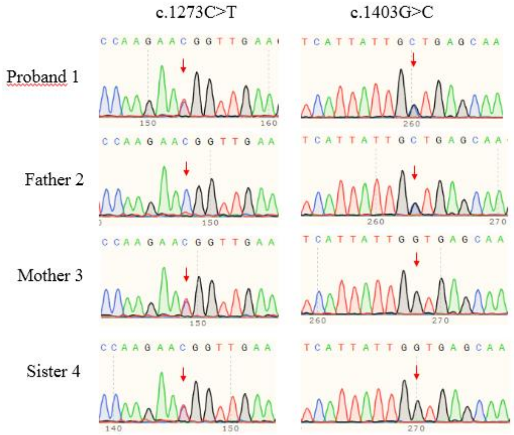

Patient ID: 3312247 *MYO15A*

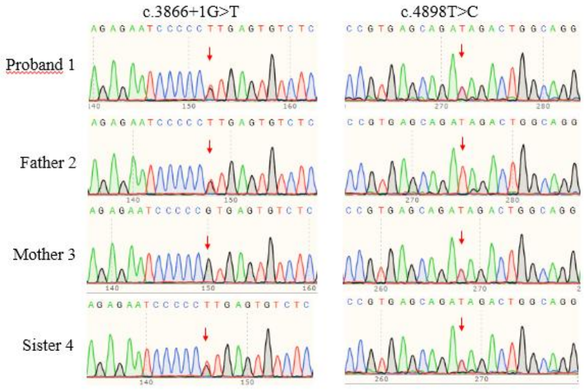

Patient ID: 3312217 *CDH23*

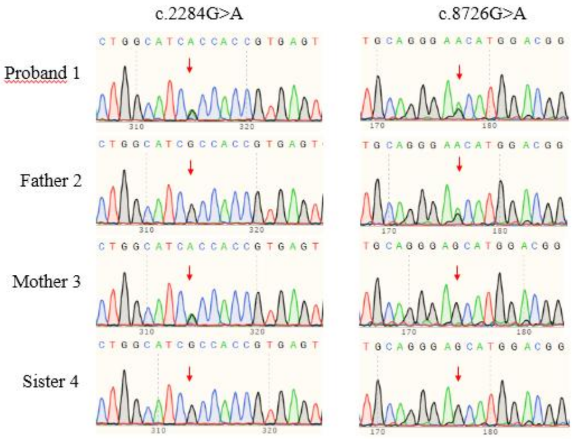

Patient ID: 3312264 *MYO15A*

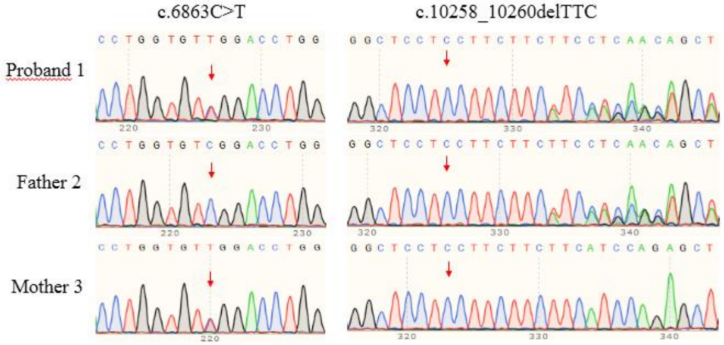

Patient ID: 3312229 *CDH23*

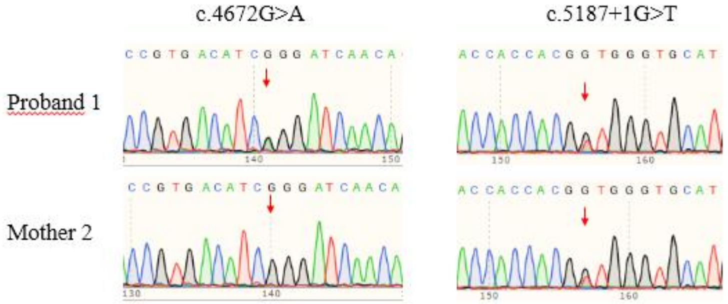

Supplement: Supplementary file 1 [file DataSheet1.PDF]
