# Supplementary material for: Increased diagnostic yield in a cohort of hearing loss families using a comprehensive stepwise strategy of molecular testing
Source: Front Genet. 2022 Dec 7;13:1057293. doi: 10.3389/fgene.2022.1057293 (PMC9768221; doi:10.3389/fgene.2022.1057293)
Supplement: Supplementary file 2 [file DataSheet2.DOCX]

Supplementary Table

# Supplementary Table S1

| Gene | Transcript | Variants | Primer F | Primer R |
| --- | --- | --- | --- | --- |
| *GJB2* | NM_004004.6 | c.-23+1G>A | CTCATGGGGGCTCAAAGGAACTAGGAGATCGG | CCCAAGGACGTGTGTTGGTCCAGCCCC |
| *MITF* | NM_198159.3 | c.1212G>A | ACCATGAAAGCTGAAGGACC | GCAGGGAGGATTCGCTAACA |
|  |  | c.1021C>T | GTACACGGCTTGGGTGGT | GTCAACTCCCCTATGGCTCA |
|  |  | c.935T>C | GCTTTTGAAAACATGCAAGC | CAGCTGTAGGAATCAACTCTCC |
| *PTPN11* | NM_002834.5 | c.923A>G | CATCAGGCAGTGTTCACGTT | GGAACACCATCCGCCAAAAG |
|  |  | c.1507G>A | GGGAATCCTGACTTCTGCCA | CTTCTTCAATCCTGCGCTGT |
| *TRIOBP* | NM_001039141.3 | c.1165A>T | CTCACGAAGCACCCAACTGGATAA | GAGGTTCTGGAGGCTCTGGGATTG |
| *MPZL2* | NM_005797.4 | c.277_285del | ACCCCAGAACTCTTCGGTCT | TGCTGGAGGCTGTTAATGGG |
|  |  | c.220C>T | GTCCTTAAACCGCCCACTCA | AGGCGAGGCTTTAATGCTGT |
| *POLD1* | NM_001256849.1 | c.3263A>G | CAGGAGCCGTGTGTGAGTT | TCACAGCTGGAAGGGGATG |
| *MYO15A* | NM_016239.4 | c.4039-2A>G | CTTGCTGTGTGGGAGTTGG | CAAAGCGGCTGGAGTTGTC |
|  |  | c.10419_10423del | ACTGGGGAACTGGTACTTGA | TGTGGTTGGTTCTGTCCCAT |
|  |  | c.8791del | TCCCCTTTATGGTCCTGTGG | CTTATCCCCACTCGCCTCAC |
|  |  | c.10250_10252del | CATGAGAAGGGAGGCCAGAG | GGAACAGAGGGTGGCGAT |
|  |  | c.4945C>T | TGTTCTGGAGGGAAAGGTGC | CTCCCAACTGAGCACCAGAG |
|  |  | c.9534dup | AGCGGTTTCCAGGCGTATAG | GGGCTTGTGTCTCCCATTCA |
|  |  | c.4109G>A | GGACAGGCAGGAAAGGACAT | TAATTTGACGTCTACCTAGCCC |
|  |  | c.6863C>T | GTACCTGGAATGTTGTGGGG | AGCTACCTACACTTTGGGGC |
|  |  | c.10258_10260del | ATGGAGGATGGGTATGGAGG | GACTTGCCTGAGATCATGAAGG |
|  |  | c.3866+1G>T | TAGGGGAGGGAGGGACATAG | CCTCCAACCTGTAGCTTCGG |
|  |  | c.4898T>C | GTTCTGGAGGGAAAGGTGCT | TGAATAGTAGGTCGGGTGTCC |
| *SOX10* | NM_006941.4 | c.91_100del | GTGGGCGTTGGACTCTTTG | CTACCCTGAATCCACCCGAA |
| *USH2A* | NM_206933.4 | c.8559-2A>G | GCCCAGAACTAAATGCCAGC | AGAACAGCCTGAGTTTTGGT |
|  |  | c.1824dup | TCAAAGTAAGCATTCCCGAGT | ACCTTGTCAATGCAACAGCC |
| *TMPRSS3* | NM_001256317.3 | c.646C>T | CTGACATGACCCAGGAGTGA | CAGGTGCATGGTGGTGAC |
|  |  | c.323-6G>A | TCCTCTCTGTGTTTTGCCCA | AGTGGGTCAGATTTGGCAGA |
| *PCDH15* | NM_001142769.3 | c.4806_4809dup | TCAACCATGGGCCTTCTTCT | CTTGAGGGTGAAGAGTGGC |
|  | NM_033056.4 | c.3300_3319dup | TGAAATCACTCCCTGCCTTG | ACAGTTTCCAAAAGCCAGCA |
| *LOXHD1* | NM_001384474.1 | c.5885C>T | CATTTGTGTACCTATGGCTCCC | GCCTCAATCGTGGTGCTTTT |
|  |  | c.2047+1G>T | CACCATTGGCCAGCATTACT | GCTTTGATTGTCTGCCTGCT |
| *POU3F4* | NM_000307.5 | c.988A>T | GACAAGATCGCTGCACAGG | CACGGTGTGCGAATAAACCT |
|  |  | c.730A>T | CACCATTGCCAGGATCACTC | GCGATCTTGTCAATGCTGGT |
| *CDH23* | NM_022124.6 | c.982G>A | TCCCTCTTGTCCCTCGATGA | TTGCAGGGTGTAGGGGATAA |
|  |  | c.8239G>A | TGTGATGTTCCTTGGCGACT | TACTCAGCCTTGGTGAAGCG |
|  |  | c.739GA>AG | GGACCTTGGGGATGCTGTTT | GGACCCTTGAAGAAGGGCAG |
|  |  | c.3878A>G | CTGGACCTTGCCATCCACAT | CATTTTATGACAAAGAGGAGAGTGC |
|  |  | c.5586G>T | CATGACCAACTGCACCCTC | GATGTTGAAGGTGAGGCGTG |
|  |  | c.7396G>A | AGTGAGAGCAAGAAGAGCCC | CCTGCACTGAATTTTCGCGA |
|  |  | c.2284G>A | TCATGGGGTTATGTTGCCCA | CTCTGAGCTGCTCTTCCTCG |
|  |  | c.8726G>A | ATGATAACCGGCTGAGACCC | AGGTCTCGGGCCACAATG |
|  |  | c.4672G>A | TCTGGGGTAGATGCAGCAAA | CGATCTCACCGCTGATGC |
|  |  | c.5187+1G>T | CAAATTGGTCAGAGGGTGCT | TCCCTCAGACAATCTTCCCC |
| *COL11A2* | NM_080680.3 | c.3312+3A>C | GGGTCATGGGTCAGGTGTTC | GATGAGATGGCTGACCTGGG |
|  |  | c.966dup | CCAACAGCCAAGGATCGAAAC | TTTGGCCTTTGATGGCCCTT |
| *HARS2* | NM_012208.4 | c.1273C>T | GAGGTGTAGTTGGAGTGGTTT | CTGGGTCCTAAATTCCACACAT |
|  |  | c.1403G>C | GTGTGGAATTTAGGACCCAGG | TCGTCCCTTCTATTTCTGCTG |

# Supplementary Table S2

Twenty-eight diagnoses/probable diagnoses made by WES. *AD* autosomal dominant, *VUS* variant of uncertain significance, *PVS* pathogenic very strong, *PS* pathogenic strong, *PM* pathogenic moderate, *PP* pathogenic supporting.

| **Patient ID** | **Gene** | **Transcript** | **Variant** | | **Zygosity** | **Inheritance** | **ACMG Classification** | **References** |
| --- | --- | --- | --- | --- | --- | --- | --- | --- |
| **Diagnoses** | | | | | | | | |
| 3312176 | *MITF* | NM_198159.3 | c.1212G>A, p.Thr404= | | Het | AD | P (PS3, PM2, PP1_Strong) | PMID: 21438779 |
| 3312205 | *PTPN11* | NM_002834.5 | c.923A>G, p.Asn308Ser | | Het | AD  (*de novo*) | P (PS2, PM2, PM5_Strong) | PMID: 11992261 |
| 3312216 | *TRIOBP* | NM_001039141.3 | c.1165A>T, p.Arg389* | | Hom | AR | P (PVS1, PM2, PM3_Supporting) | novel |
| 3312236 | *MPZL2* | NM_005797.4 | c.277_285del, p.Asp93_Val95del | | Het | AR | LP (PM2, PM3, PM4) | novel |
|  |  |  | c.220C>T, p.Gln74* | | Het |  | P (PVS1, PM3_Strong) | PMID: 29961571 |
| 3312343 | *POLD1* | NM_001256849.1 | c.3263A>G, p.Gln1088Arg | | Het | AD  (*de novo*) | LP (PS2, PM2) | SCV001477293 |
| 3312194 | *MYO15A* | NM_016239.4 | c.4039-2A>G, - | | Het | AR | P (PVS1, PM2, PM3) | novel |
|  |  |  | c.10419_10423del, p.Ser3474ProfsTer42 | | Het |  | P (PVS1, PM2, PM3) | doi:10.15761/OHNS.1000207 |
| 3312301 | *SOX10* | NM_006941.4 | c.91_100del, p.Leu31ThrfsTer75 | | Het | AD  (*de novo*) | P (PVS1, PS2, PM2) | PMID: 32234158 |
| 3312198 | *USH2A* | NM_206933.4 | c.8559-2A>G, - | | Het | AR | P (PVS1, PM2, PM3) | PMID: 10909849 25356976 19737284 26338283 |
|  |  |  | c.1824dup, p.Glu609Ter | | Het |  | P (PVS1, PM2, PM3) | PMID: 10729113 10909849  SCV001411290 |
| 3312313 | *TMPRSS3* | NM_001256317.3 | c.646C>T, p.Arg216Cys | | Het | AR | P(PM2, PM3_Strong, PM5_Strong, PP3) | PMID: 17551081 |
|  |  |  | c.323-6G>A, - | | Het |  | P(PS3, PM2, PM3_Strong, BP4) | PMID: 11137999  21786053 28695016  15447792 |
| 3312279 | *PCDH15* | NM_001142769.3 | c.4806_4809dup, p.Glu1604IlefsTer11 | | Het | AR | P(PVS1, PM2, PM3) | SCV000788418 |
|  |  | NM_033056.4 | c.3300_3319dup, p.Val1107GlyfsTer67 | | Het |  | P(PVS1, PM2, PM3) | novel |
| 3312282 | *LOXHD1* | NM_001384474.1 | c.6071C>T, p.Thr1962Met | | Het | AR | LP(PM2, PM3_Strong, PP3) | PMID: 28000701  SCV000340312  SCV000796451 |
|  |  |  | c.2047+1G>T, - | | Het |  | P(PVS1, PM2, PM3) | novel |
| 3312291 | *PTPN11* | NM_002834.5 | c.1507G>A, p.Gly503Arg | | Het | AD  (*de novo*) | P(PS1, PM5_Strong, PM2, PP3) | PMID: 23513489 |
| 3312304 | *POU3F4* | NM_000307.5 | c.988A>T, p.Arg330* | | Hemi | X-link | P(PVS1, PM2, PP4) | novel |
| 3312249 | *MITF* | NM_198159.3 | c.1021C>T, p.Arg341Cys | | Het | AD | LP(PM2, PM5, PP1, PP3) | PMID: 32728090  27057829 |
| 3312311 | *MITF* | NM_198159.3 | c.935T>C, p.Leu312Pro | | Het | AD  (*de novo*) | LP (PS2, PM2, PP3) | SCV001164284 SCV000781743 |
| 3312317 | *MYO15A* | NM_016239.4 | c.8791del, p.Trp2931fs | | Het | AR | P(PVS1, PM2, PM3) | novel |
|  |  |  | c.10250_10252del, p.Ser3417del | | Het |  | LP(PM2, PM3, PM4) | SCV001244795 |
| 3312298 | *MYO15A* | NM_016239.4 | c.4109G>A, p.Arg1370His | | Het | AR | LP(PM2, PM3, PM5, PP3) | SCV000272091 |
|  |  |  | c.10250_10252del, p.Ser3417del | | Het |  | LP(PM2, PM3, PM4) | SCV001244795 |
| **Probable diagnoses** | | | | | | | | |
| 3312167 | *CDH23* | NM_022124.6 | c.982G>A, p.Ala328Thr | | Het | AR | VUS (PM2_Supporting) | SCV000883554 |
|  |  |  | c.8239G>A, p.Val2747Met | | Het |  | VUS (PM2_Supporting) | novel |
| 3312225 | *MYO15A* | NM_016239.4 | c.4945C>T, p.Leu1649Phe | | Het | AR | VUS (PM2, PM3, PP3) | SCV000966690 |
|  |  |  | c.9534dup, p.Glu3179ArgfsTer43 | | Het |  | LP (PVS1, PM2) | novel |
| 3312234 | *CDH23* | NM_022124.6 | c.739G>A c.740A>G | p.Glu247Arg | Het | AR | VUS (PM2, PP3) | novel |
|  |  |  | c.3878A>G, p.Asn1293Ser | | Het |  | VUS(PM2) | novel |
| 3312358 | *COL11A2* | NM_080680.3 | c.3312+3A>C, - | | Het | AR | VUS (PM2, PM3) | novel |
|  |  |  | c.966dup, p.Tyr323LeufsTer4 | | Het |  | P (PVS1, PM2, PM3) | PMID: 29456477 |
| 3312206 | *CDH23* | NM_022124.6 | c.5586G>T, p.Glu1862Asp | | Het | AR | VUS(PM2) | novel |
|  |  |  | c.7396G>A, p.Glu2466Lys | | Het |  | VUS (PM2, PP3) | novel |
| 3312255 | *HARS2* | NM_012208.4 | c.1273C>T, p.Arg425Trp | | Het | AR | VUS (PM2, PP3) | novel |
|  |  |  | c.1403G>C, p.Gly468Ala | | Het |  | VUS (PM2, PP3) | novel |
| 3312264 | *MYO15A* | NM_016239.4 | c.6863C>T, p.Ser2288Leu | | Het | AR | VUS (PM2) | SCV000401173  SCV000712122 |
|  |  |  | c.10258_10260del, p.Phe3420del | | Het |  | VUS (PM2, PM4) | novel |
| 3312302 | *POU3F4* | NM_000307.5 | c.730A>T, p.Asn244Tyr | | Hemi | X-link | VUS(PM2, PP3, PP4) | novel |
| 3312217 | *CDH23* | NM_022124.6 | c.2284G>A, p.Ala762Thr | | Het | AR | VUS(PM2) | novel |
|  |  |  | c.8726G>A, p.Ser2909Asn | | Het |  | VUS(PM2, BP4) | SCV000062972  SCV001565014 |
| 3312229 | *CDH23* | NM_022124.6 | c.4672G>A, p.Gly1558Arg | | Het | AR | VUS(PM2, PM3, PP3) | novel |
|  |  |  | c.5187+1G>T, - | | Het |  | LP(PVS1, PM2) | SCV001240893  SCV001574062 |
| 3312247 | *MYO15A* | NM_016239.4 | c.3866+1G>T, - | | Het | AR | LP(PVS1, PM2) | novel |
|  |  |  | c.4898T>C, p.Ile1633Thr | | Het |  | VUS(PM2, PM3, PP3) | PMID: 24853665 |
